# Prognostic Significance of Left Ventricular Fibrosis Assessed by T1 Mapping in Patients with Atrial Fibrillation and Heart Failure

**DOI:** 10.1038/s41598-019-49793-8

**Published:** 2019-09-16

**Authors:** Lei Zhao, Songnan Li, Xiaohai Ma, Rong Bai, Nian Liu, Ning Li, Paul Schoenhagen, Changsheng Ma

**Affiliations:** 1 Department of Radiology, Beijing Anzhen Hospital, Capital Medical University, Beijing, China; 2 Department of Cardiology, Beijing Anzhen Hospital, Capital Medical University, Beijing, China; 3 Department of Intervention, Beijing Anzhen Hospital, Capital Medical University, Beijing, China; 4 Imaging Institute and Heart & Vascular Institute, Cleveland Clinic Lerner College of Medicine, Cleveland, Ohio USA

**Keywords:** Cardiology, Cardiovascular diseases

## Abstract

This study sought to investigate whether left ventricular (LV) fibrosis quantified by T1 mapping can be used as a biomarker to predict outcome in patients with atrial fibrillation (AF) and heart failure (HF). 108 patients with AF and HF were included in this study. They underwent cardiac magnetic resonance, including T1 mapping sequence to assess LV fibrosis between May 2014 to May 2016. Patients received catheter ablation for AF and pharmacological treatment for HF. The primary endpoint was a composite adverse outcome of cardiac death, subsequent HF or stroke, subsequent HF was the secondary endpoint. During follow up (median: 23 months, Q1-Q3: 11 to 28 months), 1 cardiac death, 12 strokes, and 42 HF episodes occurred. LV extracellular volume fraction (ECV) was predictive of composite adverse outcome and subsequent HF (all p < 0.001). In multivariable analysis, LV ECV was an independent predictor of composite adverse outcome (hazard ratio (HR): 1.258, 95% confidence interval (CI): 1.140–1.388, p < 0.001) and subsequent HF (HR: 1.223, 95% CI: 1.098–1.363, p < 0.001). LV fibrosis measured by T1 mapping indices significantly predicts composite adverse outcomes and subsequent HF in patients with AF and HF.

## Introduction

Atrial fibrillation (AF) and heart failure (HF) are increasingly prevalent and frequently coexist^1–3^. When present in combination, the prognosis appears to be worse^4^. However, the predictors of adverse outcome in this cohort remain unclear. In patients with AF, left ventricular (LV) fibrosis has been demonstrated by histopathology and noninvasive imaging^5,6^. Similarly, in patients with HF, LV fibrosis plays an essential pathophysiological role^7–9^. Although its clinical significance in HF and AF is not fully understood, LV fibrosis represents a potential link between AF and HF^10^. Assessing the degree of LV fibrosis may provide information for predicting outcomes in patients with coexisting AF and HF.

Ventricular myocardial fibrosis includes replacement fibrosis and interstitial fibrosis. Using late gadolinium enhancement (LGE) sequence, cardiovascular magnetic resonance (CMR) provides excellent visualization of replacement fibrosis. Interstitial fibrosis is difficult to distinguish by LGE because it is extensively distributed, the myocardial signal intensity may be nearly isointense which is hard to differentiate from normal tissues^11^. Recent innovations in CMR permit quantitative measurements of both replacement fibrosis and interstitial fibrosis using T1 mapping techniques regardless of its distribution^12,13^. The detection and quantification of LV fibrosis with T1 mapping have been validated against collagen content in subjects with a variety of cardiomyopathies^14,15^. T1 mapping method including two important indices: 1) native T1 reflects myocardial abnormality involving the myocyte and interstitium; 2) after use of gadolinium-based contrast agents, extracellular volume fraction (ECV) measures the extent of the extracellular space, reflecting myocyte loss and interstitial disease^16^. Suksaranjit *et al*. showed the prognostic impact of LV replacement fibrosis assessed by LGE in patients with AF^17^. However, the clinical value of T1 mapping for identification of LV fibrosis in patients with coexisting AF and HF is still unclear.

We hypothesized that quantification of LV fibrosis with T1 mapping would predict adverse outcome in patients with AF and HF. The purpose of our study was to assess LV fibrosis by determining the ranges of T1 mapping indices in patients with AF and HF, and investigate whether T1 mapping could be used as a quantitative imaging biomarker to predict the adverse outcome.

## Materials and Methods

### Study design

We performed a prospective observational study of patients with coexisting AF and HF who admitted to our hospital for AF catheter ablation between May 2014 to May 2016. All patients had ECG documented AF, they were symptomatic, and refractory to at least one antiarrhythmic drug. Presence and severity of HF were ascertained according to 2016 ESC Guidelines for the diagnosis and treatment of acute and chronic HF, and classified as HF with preserved ejection fraction (HFpEF, LVEF ≥ 50%), HF with mid-range EF (HFmrEF, LVEF 40%-49%), and HF with reduced EF (HFrEF, LVEF < 40%)^18^. We excluded patients with valvular heart disease, acute myocardial infarction, myocarditis, evidence of infiltrative cardiomyopathy, and severe impairment of renal function (glomerular filtration rate <30 ml/min/1.73 m^2^). Finally, 108 patients with AF and HF who underwent CMR with T1 mapping in our hospital prior to AF catheter ablation were included in this study. All underwent catheter ablation for AF and adjustment of pharmacological treatment for HF prior to discharge. Thorough clinical data were obtained by reviewing medical records, physical examination, and routine lab tests. Follow up was performed by telephone interview of the patients, their general practitioner/cardiologist and/or family members. The primary study endpoint was the composite of cardiac death, stroke, and HF readmission. The second study endpoint was HF readmission. In addition, recurrence of AF after ablation (defined as any AF episode >30 seconds identified by12-lead ECG or on repeat Holter monitoring after 3 months of blank period) were recorded. Complete follow-up was obtained for all patients. The T1 mapping indices of 49 gender and age-matched healthy volunteers scanned at our site with the same MR system were included as the reference standard. The investigation conformed with the principles outlined in the Declaration of Helsinki. The study was approved by the Beijing Anzhen Hospital ethical committee, and written informed consent was obtained from all patients.

### CMR examination

All CMR exams were performed using a 3T MR system (MAGNETOM Verio, Siemens Healthcare) with a 32-channel cardiac coil. Steady-state free-precession cine images were obtained during repeated breath-holding in two long-axis views (two chambers and four chambers) and in a stack of short-axis views covering the LV for quantification of cardiac chamber volumes and function. In the case of AF episodes during the exam, the acquisition was performed with a 2-dimensional (2D) real-time true fast imaging sequence with steady precession during a single breath-hold. Late gadolinium enhancement (LGE) imaging was performed in the same planes as cine imaging using a phase-sensitive inversion-recovery sequence approximately 10 minutes after administration of 0.1 mmol/kg gadopentetate dimeglumine.

T1 mapping was obtained using a modified look-locker inversion recovery sequence (MOLLI). Data were acquired in basal, mid-ventricular, and apical short-axis planes before and 15 min after contrast administration. T1 mapping images were acquired in the systolic phase (trigger delay = 0 ms) with a heart-rate-dependent pulse sequence sampling scheme as previously described in order to eliminate the impact of the irregular and usually rapid ventricular rate of AF (the number of inversions (n) was determined by referencing the highest heart rate before the actual scan, 5(n)3 and 4(n)3(n)2 for precontrast and postcontrast T1 mapping, respectively)^19^. Imaging parameters were TR = 2.6–2.7 ms, TE = 1.0–1.1 ms, FA = 35°, FOV = 270 × 360 mm^2^, matrix 256 for heart rate <90 bpm, 192 for heart rate ≥90 bpm, BW = 1045–1028 Hz/px, GRAPPA acceleration factor 2, minimum TI = 120 ms. Quality control was performed during scanning by reviewing the “goodness of fit” map and source images to allow an immediate repetition of suboptimal measurements to minimize the respiratory motion and off-resonance effects. A blood sample was taken just before the CMR exam to measure haematocrit for the ECV calculation.

All CMR images were analysed by a radiologist who was blinded to clinical and study participant information. Both the LV systolic and diastolic function were assessed. Global LV functional indices were analysed using dedicated software (Argus, Siemens Healthcare, Erlangen, Germany). The following indices were measured: LV EF, LV end-diastolic volume (EDV), LV end-systolic volume (ESV), LV stroke volume (SV) and LV mass. Except for LV EF, all parameters were adjusted by body surface area (BSA).

2D cardiac performance analysis software (QMass, Medis, Leiden, the Netherlands) was used to obtain LV peak systolic circumferential strain (ε_s_), peak systolic circumferential strain rate (SR_s_), and peak diastolic circumferential strain rate (SR_e_) data directly from cine mid-ventricular short-axis view images, as previously described^20^. Left atrium (LA) volume was measured at end-systole using the biplane area-length method. LA volume and SR_e_ were used as LV diastolic functional indices.

All LGE and T1 mapping image datasets were transferred to Syngo workstation (Siemens Healthcare, Erlangen, Germany) for offline analysis. LV replacement fibrosis was identified qualitatively by LGE within the myocardium; it was considered present only if confirmed on both short-axis and matching long-axis myocardial locations. Then the quantitative measurement of LV fibrosis was performed with native T1 and ECV. The LV myocardium was delineated by manually contouring the endocardial and epicardial borders of precontrast and postcontrast T1 maps. According to the AHA 16-segment model, segments with artefact were excluded from the LV myocardium delineation. The overall LV myocardial native T1 time was the mean of myocardial T1 times of the basal, mid-ventricular and apical levels on precontrast T1 maps. The overall LV ECV was calculated from precontrast and postcontrast T1 mapping images that were calibrated by blood haematocrit^19,21^. To assess the interobserver agreement, all T1 mapping images were reanalysed by a second experienced and blinded radiologist.

### Catheter ablation

All antiarrhythmic drugs were stopped before catheter ablation. After transseptal puncture under sedation, a bolus of intravenous heparin (100 U/kg) was administered. During the procedure, an activated clotting time of >300 s was maintained. A 3.5 mm open-irrigation ablation catheter (NAVISTAR THERMOCOOL, Biosense-Webster, CA, USA) was advanced into the left atrium for mapping and ablation with a 3D electroanatomical mapping system (CARTO 3, Biosense-Webster, CA, USA). For patients with PAF, pulmonary vein electrical isolation was achieved by continuous circumferential pulmonary vein ablation. For persistent AF patients, we also use a fixed strategy called “2C3L” including PV isolation and linear ablation across mitral annulus, LA roof and tricuspid isthmus^22^. The procedural endpoint was pulmonary vein isolation and block of all ablated lines which was achieved in all the cases. The vascular access-site hematoma was observed in 2 cases, which were resolved conservatively.

### Statistics

The analysis was conducted using SPSS software (version 21, IBM, Armonk, NY, USA). Normality of data was assessed with Kolmogorov-Smirnov tests. All data are expressed as the means ± standard deviation (SD) unless otherwise indicated. Comparisons between different HF groups were made using 1-way analysis of variance or Kruskal Wallis test for continuous variables and chi-square test for categorical variables, as appropriate. Univariate Cox proportional hazards model was used to test the association between baseline covariates and primary endpoint, between baseline covariates and secondary endpoint respectively. Multivariable analysis was performed with a selection of variables with p < 0.1 to enter the model. Event-free survival was determined according to the Kaplan-Meier method using the median ECV value^7,23^, and comparison of survival rate was performed using a log-rank test. The reference value was derived from 49 healthy volunteers (19 females, age 55 ± 12 years) who were scanned in our site using the same MR scanner, LV native T1 _average_ = 1251 ms ± 36; LV ECV _average_ = 25.7% ± 2.4. The interobserver agreement was assessed using the intraclass correlation coefficient. A p-value of <0.05 was considered significant, and all reported p values are 2-tailed. The sample size was calculated on the basis of a proposed difference of 3.0% in absolute ECV values between patients with and without composite adverse outcomes, a standard deviation of 2.5% for ECV measurements. To identify this difference with a power of 80% (alpha = 0.05), 9 patients in each group (with vs. without events) were needed.

## Results

**Figure 1:**
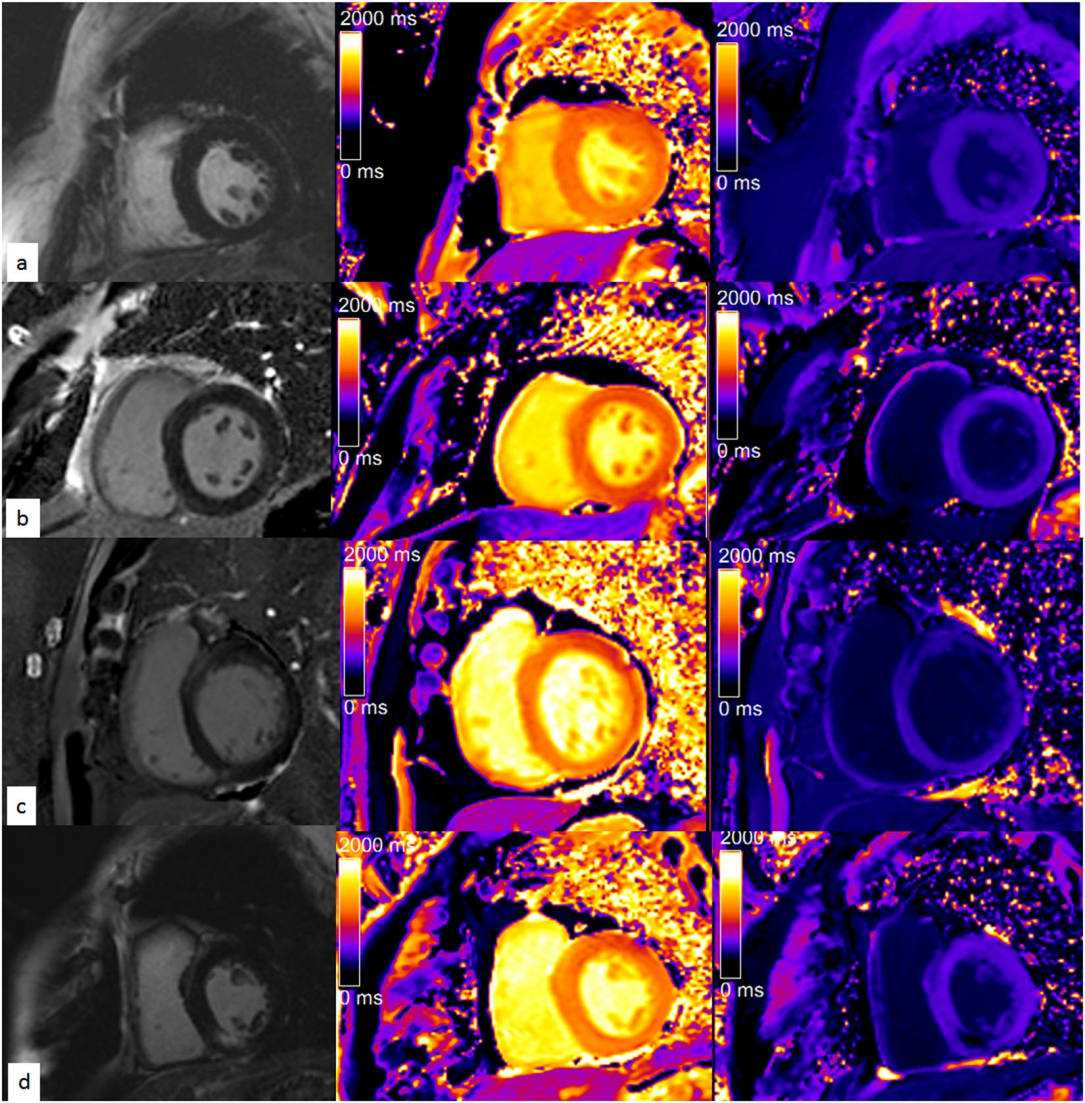
Representative T1 mapping images. (**a**) A 48-year-old woman with persistent AF and hypertension and HFrEF. The image shows an apparent normal myocardium without evidence of late gadolinium enhancement (LGE) on the standard visual assessment; the left ventricular (LV) mean native T1 time is 1295 ms and postcontrast T1 time is 567 ms at mid-ventricular level, the ECV is 27.1%. (**b**) A 51-year-old man with paroxysmal AF and HFrEF. The image shows an apparent normal myocardium without evidence of LGE; the LV mean native T1 time is 1271 ms, and the postcontrast T1 time is 621 ms at the mid-ventricular level, the ECV is 27.4%. (**c**) A 75-year-old man with persistent AF, hypertension, and an HFrEF. The image shows LGE at the anterolateral segment; the LV mean native T1 time is 1311 ms, and the postcontrast T1 time is 453 ms at the basal level, the ECV is 31.4%. (**d**) A 65-year-old man with paroxysmal AF, diabetes, hypertension, and HFpEF. The image shows LGE at the inferoseptal segment; the LV mean native T1 time is 1300 ms, and the postcontrast T1 time is 538 ms at mid-ventricular level, the ECV is 30.0%.

The baseline general demographic data and imaging characteristics of patients are presented in Table 1, Table 2 and Fig. 1. Based on qualitative assessment, LGE was detected in 36% of the 108 included patients (n = 39). The LGE pattern was ischemic in 6 patients (subendocardial enhancement). In the remaining 33 patients a non-ischemic LGE pattern was identified, including 4 patients with a pattern consistent with hypertrophic cardiomyopathy (2 patients with HFpEF and apical hypertrophic cardiomyopathy, with slightly diffuse LGE in LV apical level myocardium; 1 patient with HFmrEF, 1 patient with HFrEF, both patients with asymmetric hypertrophic cardiomyopathy and focal LGE at RV insertion points), and 29 patients with a non-specific LGE pattern (most LGEs were located at mid-wall of basal septal myocardium or mid-wall of inferior-lateral myocardium, microembolism cannot be ruled out).

**Table 1:** Baseline Patient Characteristics (n = 108).

Characteristic	HFpEF (n = 55)	HFrEF (n = 18)	HFmrEF (n = 35)	p Value
Age, years	56 ± 14	54 ± 7	55 ± 10	0.880
Female	10	3	7	0.058
Height, cm	1.7 ± 0.1	1.7 ± 0.1	1.7 ± 0.1	0.973
Weight, kg	76.8 ± 13.0	82.1 ± 12.0	77.6 ± 12.5	0.341
Body mass index, kg/m^2^	26.1 ± 3.2	27.6 ± 3.5	26.2 ± 3.4	0.235
Paroxysmal AF	39	6	14	0.002
Persistent AF	16	12	21	0.002
Duration of AF, months (median, IQR)	24, 12–84	18, 5–62	24, 6–120	0.331
NYHA grade (mean ± SD)	2.7 ± 0.8	3.1 ± 0.8	2.7 ± 0.8	<0.001
II	30	5	18	
III	13	6	9	
IV	12	7	8	
Diabetes mellitus	3	4	4	0.119
Hypertension	10	7	13	0.076
Hypercholesterolemia	14	5	7	0.775
Alcohol excess	14	8	10	0.305
Smoking	24	7	16	0.893
OSAHS	1	1	3	0.324
CHA_2_DS_2_-VaSc score	1.3 ± 1.1	1.6 ± 1.2	1.4 ± 1.3	0.595
ACE inhibitor or ARB	10	18	22	<0.001
Beta-blocker	11	5	12	0.315
Calcium-channel blocker	11	4	9	0.817
Diuretics	3	18	11	<0.001
Statin	15	4	8	0.856
Warfarin	22	9	18	0.518
NOAC	22	9	16	0.721
Class I anti-arrhythmic	15	10	18	0.024
Class III anti-arrhythmic	6	6	12	0.016

Values are expressed as the means ± standard deviation or n (%) unless otherwise indicated. HFpEF, heart failure with preserved ejection fraction (EF ≥ 50%); HFmrEF, heart failure with mid-range fraction (50% < EF ≤ 40%); HFrEF, heart failure with reduced ejection fraction (EF < 40%); NYHA, New York Heart Association; OSAHS, obstructive sleep apnoea-hypopnea syndrome; ACE, angiotensin-converting enzyme; ARB, angiotensin receptor blocker; NOAC, novel oral anticoagulants for atrial fibrillation; IQR, interquartile range. Excess alcohol defined as ≥ 8 drinks/week; Smoking, all are current smokers, no patient of quit smoking was documented.

**Table 2:** Echocardiographic and Cardiac Magnetic Resonance Characteristics.

Characteristic	HFpEF (n = 55)	HFrEF (n = 18)	HFmrEF (n = 35)	p Value
**Echocardiography**				
LV EF, %	61 ± 5	36 ± 13	48 ± 4	<0.001
LV diastolic dimension, cm	4.8 ± 0.5	5.6 ± 0.7	4.9 ± 0.4	<0.001
LA dimension, cm	4.0 ± 0.7	4.9 ± 0.7	4.4 ± 0.7	<0.001
Estimated PASP, mmHg	29.5 ± 7.4	31.8 ± 8.2	28.7 ± 5.8	0.464
**CMR**				
Body surface area, m^2^	1.9 ± 0.2	1.9 ± 0.2	1.9 ± 0.2	0.008
LA volume, ml	138 ± 39	198 ± 29	172 ± 52	<0.001
LA volume index, ml/m^2^	73.6 ± 20.2	105 ± 20	92 ± 25	<0.001
LV EF, %	60 ± 7	32 ± 6	46 ± 3	<0.001
LV EDV, ml	91 ± 25	128 ± 35	95 ± 38	<0.001
LV EDV index, ml/m^2^	48.7 ± 12.4	67.9 ± 22.1	50.1 ± 18.9	<0.001
LV ESV, ml	36 ± 11	88 ± 27	52 ± 21	<0.001
LV ESV index, ml/m^2^	20.1 ± 7.3	45.8 ± 17.8	27.2 ± 10.6	<0.001
LV SV, ml	55 ± 17	41 ± 12	43 ± 17	0.001
LV SV index, ml/m^2^	28.6 ± 9.3	22.1 ± 5.8	22.9 ± 8.4	0.002
LV mass, g	91 ± 23	130 ± 35	103 ± 27	<0.001
LV mass index, g/m^2^	48.4 ± 9.6	69.5 ± 17.9	54.1 ± 11.7	<0.001
ε_s_, %	−17.7 ± 1.2	−13.4 ± 1.2	−15.7 ± 0.8	<0.001
SR_s_, s^−1^	−91.9 ± 8.5	−62.9 ± 7.8	−78.0 ± 7.8	<0.001
SR_e_, s^−1^	99.7 ± 11.0	64.4 ± 6.0	79.9 ± 7.1	<0.001
LGE, no. of patients	15	10	14	0.080
LV native T1 _average_, ms	1275 ± 34	1349 ± 53	1302 ± 31	<0.001
LV ECV _average_, %	27.2 ± 2.3	32.3 ± 4.9	29.1 ± 2.8	<0.001

Values are expressed as the means ± standard deviation or n (%) unless otherwise indicated. HFpEF, heart failure with preserved ejection fraction (EF ≥ 50%); HFmrEF, heart failure with mid-range fraction (50% < EF ≤ 40%); HFrEF, heart failure with reduced ejection fraction (EF < 40%); PASP, pulmonary artery systolic pressure, LA dimension, left atrial anterior-posterior dimension; E/A ratio, ratio of early transmitral filling to late transmitral filling.

### LV T1 mapping indices

In the 108 patients, 42 segments in 8 pre-contrast T1 maps and 59 segments in 11 post-contrast T1 maps were excluded due to the presence of artefacts (most artefacts were presented in the subepicardial of anterior segment or inferior-lateral segment). LV mean native T1 time and ECV of all patients are presented in Table 2. When compared between patients with different AF patterns, patients with persistent AF (LV native T1 _average_ = 1305 ms ± 46; LV ECV _average_ = 29.8% ± 3.8) had a greater LV mean native T1 time and ECV than patients with paroxysmal AF (LV native T1 _average_ = 1289 ms ± 44, LV ECV _average_ = 27.7% ± 3.0, all p* < *0.01). When patients were compared according to the HF types, patients with HFrEF (LV native T1 _average_ = 1349 ms ± 53; LV ECV _average_ = 32.3% ± 4.9) had more severe LV fibrosis than patients with HFmrEF (LV native T1 _average_ = 1302 ms ± 31; LV ECV _average_ = 29.1% ± 2.8) and patients with HFpEF (LV native T1 _average_ = 1275 ms ± 34; LV ECV _average_ = 27.2% ± 2.3, all p < 0.001).

High levels of inter-observer agreement were achieved for pre-contrast T1 (intraclass correlation coefficient (ICC), 0.958; 95% confidence interval (CI): 0.939, 0.971), post-contrast T1 (ICC, 0.929; 95% CI: 0.898, 0.951).

### Adverse outcomes

55 adverse outcomes were observed in 51 patients including 1 cardiac death, 12 strokes (10 ischemic strokes, 2 haemorrhagic strokes; among these 12 stroke patients, 8 patients had history of stroke), and 42 subsequent episodes of acute decompensated HF over a median follow up period of 23 months (Q1–Q3: 11 to 28 months). This included 23 events (17 HF readmission and 6 stroke) in 22 patients with paroxysmal AF, 32 events (1 cardiac death, 25 HF readmission, and 6 stroke) in 29 patients with persistent AF; 19 events (11 HF readmission and 8 stroke) in 18 patients with HFpEF, 20 events (18 HF readmission, and 2 stroke) in 19 patients with HFmrEF, and 16 events (1 cardiac death, 13 HF readmission, and 2 stroke) in 14 patients with HFrEF.

The clinical and imaging characteristic of patients with and without composite endpoint events are presented in Table 3. Patients with endpoint events were more likely to have persistent AF, HFrEF, decreased systolic (LV EF) and diastolic (LA volume index, SR_e_) function, and greater LV native T1 time and ECV. In addition, LV native T1 time and ECV were significantly greater in patients with HF readmission compared to those without HF readmission (1328 ms ± 40 vs. 1276 ms ± 36, 30.9% ± 3.4 vs. 27.2% ± 2.7, all p < 0.001), respectively. The LV native T1 time and ECV of 12 patients with subsequent stroke were 1299 ms ± 51, 29.6% ± 4.0 respectively.

**Table 3:** Clinical and Imaging Characteristic Between Patients With and Without Composite Adverse Outcomes.

	Patients without composite adverse outcomes (n = 57)	Patients with composite adverse outcomes (n = 51)	p Value
Age, years	54 ± 13	57 ± 10	0.163
Female	8	12	0.205
Body mass index, kg/m^2^	26.5 ± 3.1	26.3 ± 3.6	0.775
Paroxysmal/Persistent AF	37/20	22/29	0.023
Duration of AF, months (median, IQR)	24, 9–60	36, 5–84	0.709
CHA_2_DS_2_-VaSc score	1.0 ± 0.7	2.0 ± 1.5	<0.001
HFpEF/HFmrEF/HFrEF	37/16/4	18/19/14	0.002
Diabetes mellitus	4	7	0.250
Hypertension	13	17	0.223
LV EF, %	54 ± 11	47 ± 12	0.001
LA volume index, ml/m^2^	74.2 ± 22.5	96.4 ± 21.7	<0.001
SR_e_, s^−1^	91.6 ± 16.1	82.7 ± 15.5	0.005
LGE, no. of patients	16	23	0.066
LV native T1 _average_, ms	1274 ± 32	1321 ± 45	<0.001
LV ECV _average_, %	26.8 ± 2.1	30.7 ± 3.6	<0.001

### Predictive associations

**Figure 2:**
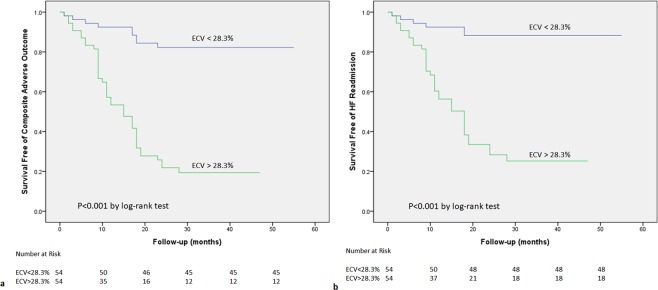
Kaplan-Meier Curve Showing Survival Free of Composite Adverse Outcome and HF Readmission in Patients Separated According to the Median ECV of 28.3%. Time is recorded in months. HF, heart failure; ECV, extracellular volume fraction. (**a**) Kaplan-Meier Curve Showing Survival Free of Composite Adverse Outcome. (**b**) Kaplan-Meier Curve Showing Survival Free of HF Readmission.

In univariate Cox regression analyses, gender, LA volume index, SR_e_, LV EF, and LV ECV showed significant predictive associations with composite adverse outcome (p < 0.05). AF patterns, CHA_2_DS_2_-VaSc score, LGE were less strongly associated with composite adverse outcome (p < 0.1, Table 4). LGE was associated with HF readmission in the univariate analysis (p < 0.05, Table 5). In multivariate analyses, gender and LV ECV were independently associated with composite adverse outcome and HF readmission respectively (Tables 4 and 5). LA volume index was also an independent predictor of HF readmission. When LV ECV increased by 2%, it was associated with 58.2%/49.6% increase in the risk of the composite adverse outcome and HF readmission respectively. Kaplan-Meier curves were generated for comparison of event-free survival from the composite adverse outcome and HF readmission according to the median ECV of 28.3% (Fig. 2). There were significant differences in event-free survival between high and low LV ECV (all p < 0.001). When only included patients with interstitial fibrosis (without LGE), in univariate Cox regression analyses, diabetes, LA volume index, SR_e_, LV EF, and LV ECV showed significant predictive associations with composite adverse outcome and HF readmission (all p < 0.05). In multivariate analyses, only LV ECV was independently associated with composite adverse outcome and HF readmission respectively (Supplementary Tables S1 and S2). In addition, 34 patients have recurrence of AF after ablation, LV native T1 time and ECV were significantly greater in patients with recurrence of AF compared to patients without recurrence of AF (LV native T1 time: 1322 ms ± 51 vs. 1282 ms ± 35, p < 0.001; LV ECV: 31.5% ± 3.9 vs. 27.1% ± 2.2, p = 0.005). LV ECV (as continuous variable with per 1% increase) was associated with recurrence of AF (HR (95% CI): 1.161 (1.047–1.288), p = 0.005).

**Table 4:** Univariable and Multivariable Analysis of Variables Predicting Composite Adverse Outcome.

Variable	Univariate Analysis Unadjusted HR (95% CI)	p Value	Multivariable Analysis Adjusted HR (95% CI)	p Value
Age	1.016 (0.991–1.042)	0.204		
Female	1.977 (1.002–3.898)	0.049	2.287 (1.042–5.021)	0.039
BMI	0.989 (0.905–1.081)	0.808		
Duration of AF	1.001 (0.996–1.006)	0.776		
AF patterns	1.707 (0.954–3.056)	0.072	0.670 (0.304–1.481)	0.323
Hypertension	1.417 (0.764–2.629)	0.269		
Diabetes mellitus	1.369 (0.616–3.042)	0.441		
CHA_2_DS_2_-VaSc score	1.224 (0.986–1.519)	0.066	1.162 (0.895–1.509)	0.260
LA volume index	1.027 (1.015–1.039)	<0.001	1.015 (0.999–1.032)	0.070
SR_e_	0.974 (0.957–0.992)	0.006	1.002 (0.955–1.051)	0.942
LV EF	0.962 (0.939–0.985)	0.002	0.990 (0.922–1.063)	0.786
LGE	1.774 (0.988–3.185)	0.055	1.453 (0.707–2.988)	0.309
LV ECV _average_ (per 1% increase)	1.315 (1.217–1.422)	<0.001	1.258 (1.140–1.388)	<0.001

**Table 5:** Univariable and Multivariable Analysis of Variables Predicting Heart Failure Readmission.

Variable	Univariate Analysis Unadjusted HR (95% CI)	p Value	Multivariable Analysis Adjusted HR (95% CI)	p Value
Age	1.010 (0.983–1.038)	0.480		
Female	2.289 (1.106–4.738)	0.026	3.228 (1.395–7.472)	0.006
BMI	0.970 (0.879–1.071)	0.548		
Duration of AF	1.001 (0.996–1.006)	0.709		
AF patterns	1.840 (0.958–3.534)	0.067	0.648 (0.273–1.538)	0.325
Hypertension	1.223 (0.603–2.479)	0.577		
Diabetes mellitus	1.147 (0.450–2.921)	0.774		
LA volume index	1.033 (1.019–1.047)	<0.001	1.020 (1.002–1.039)	0.027
SR_e_	0.964 (0.944–0.985)	0.001	1.007 (0.953–1.063)	0.810
LV EF	0.949 (0.924–0.975)	<0.001	0.971 (0.899–1.050)	0.462
LGE	1.919 (1.003–3.672)	0.049	1.904 (0.868–4.177)	0.108
LV ECV _average_ (per 1% increase)	1.312 (1.207–1.427)	<0.001	1.223 (1.098–1.363)	<0.001

## Discussion

Our results demonstrate that noninvasive quantification of LV fibrosis with T1 mapping techniques is predictive of composite adverse outcome and subsequent HF exacerbation in patients with coexisting AF and HF. Specifically, in multivariate analyses, LV ECV was an independent predictor of composite adverse outcome and HF readmission. These results demonstrated that LV fibrosis is associated with adverse outcome in patients with coexisting AF and HF and further investigation is needed to validate whether LV ECV can be used as a marker of risk stratifying in this patient population. It’s worth noting that we enrolled patients with coexisting AF and HF, where the onset of one condition compared with the other is unclear. However, in a large, community-based cohort study which investigated the temporal association between AF and HF, Santhanakrishnan *et al*. reported that AF more likely antedates to rather than follow HF^4^. Further investigation with clear precedence order of these two conditions is needed to determine whether LV fibrosis or other potential underlying mechanisms including autonomic changes and/or ion current remodelling provide a mechanistic link between AF and HF.

AF and HF frequently coexist and together adversely affect patient prognosis, yet evidence-based evaluation of this patient group is lacking^2^. In patients with composite adverse outcome, more severe baseline HFrEF, persistent AF, lower EF, larger LA volume, and advanced T1 mapping indices were present. Similar to what the previous study reported, our results showed that patients with persistent AF had lower EF than paroxysmal AF^6^. The potential explanation for more HFrEF in persistent AF patients may originate from different AF burden between persistent AF and paroxysmal AF. Advanced AF burden (such as persistent AF) may have a more obvious adverse impact on cardiac function. The prognostic impact of LV fibrosis, which is considered a common pathophysiologic process shared by AF and HF, has been suggested by prior studies, focusing on the patient with either AF or HF. In a larger study, Neilan *et al*. showed that LV replacement fibrosis detected by LGE is a frequent finding in AF predicting mortality^24^. Ling *et al*. demonstrated that LV diffuse fibrosis assessed by T1 mapping is associated with systolic dysfunction in patients with AF^6^. LV fibrosis assessed by LGE or T1 mapping indices also predicts recurrent AF after ablation^23,25,26^. LV LGE was detected in 39 patients (36%) in our study. Different from previous studies, LV LGE wasn’t associated with the composite adverse outcome in multivariate analysis. The potential explanation is the relatively small extent of LGE in our patients weakened its effect in predicting adverse outcomes, and some small extent subendocardial LGE may be missed by conventional LGE sequence^27^. LV fibrosis quantified by T1 mapping indices is associated with adverse outcomes across the spectrum of aetiologies and stages of HF^7,8,28^. However, to the best of our knowledge, this is the first report on the association between adverse outcome and T1 mapping indices in patients with coexisting AF and HF, demonstrating the clinical relevance of LV fibrosis in this population. In patients with coexisting AF and HF, our results demonstrate that LV fibrosis measured by T1 mapping indices (interstitial fibrosis + replacement fibrosis or interstitial fibrosis alone) is strongly associated with adverse outcomes and can be used as an independent predictor.

In patients with AF, atrial fibrosis identified by LGE was independently associated with recurrent arrhythmia after catheter ablation and increased risk of stroke from a series of CAMERA-MRI trials^29,30^. However, identification and analysis of atrial fibrosis remain challenging at most of the centers with current technique and available software. In fact, atrial and ventricular fibrosis may develop and progress simultaneously and to a similar extent under many situations, given the difficulty in the quantitative assessment of atrial fibrosis, whether we could use ventricular fibrosis as a surrogate of atrial fibrosis warrants further study in AF patients. Comparing to general AF population, our results showed that LV ECV was associated with recurrence of AF in patients with AF and HF. Indeed, patients with AF and HF can benefit from catheter ablation which leads to lower rates of death and HF readmission^31,32^. It is worth noting that the association between severity of LV fibrosis and recurrence of AF in this population. Whether there are differences in the incidence and impact of AF recurrence on AF patients with and without HF remain unclear and need further investigation.

In the present study, the multivariable model showed that female gender was associated with composite adverse outcome and subsequent HF in patients with AF and HF. Interpretation is limited because only 19% of patients in our study population was female. However, in prior studies that demonstrated an association between severe LA fibrosis and increased major adverse cardiovascular and cerebrovascular events in AF patients, female patients were more prevalent in the advanced LA fibrosis group than the mild LA fibrosis group (51.7% vs. 35.6%)^29^. Suksaranjit *et al*. reported a negative association between male gender (HR: 0.63, 95% CI: 0.47–0.86) and adverse outcome^26^. The association of female gender and adverse outcomes in patients with AF and HF need to be verified by further studies.

Our study has several limitations. First, we did not exclude patients with common concomitant diseases (such as diabetes mellitus and hypertension), all these comorbidities to a certain degree contribute to the development of LV fibrosis, but this study was not specifically powered to evaluate the aetiology of LV fibrosis. Second, we included patients who underwent catheter ablation for AF, adjustment of pharmacological treatment for HF were at the discretion of the treating physician. There was potential for selection bias which may have influenced clinical outcome. Third, some patients experienced AF episodes during the CMR exam, real-time cine sequence and motion-corrected LGE sequence were used^33^. Real-time cine sequence results in a slight overestimation of the ESV and subsequent underestimation of EF; however, differences in the values were in the reasonable range^34^. Fourth, we used a 16-segment model for fibrosis assessment. The 17^th^ segment was too thin to accurately assess due to the limited spatial resolution.

In summary, LV fibrosis quantified by T1 mapping indices could potentially predict composite adverse outcome and subsequent HF in patients with coexisting AF and HF. The predictive associations support the independent clinical relevance of LV fibrosis in patients with coexisting AF and HF. Our findings identify the value of noninvasive quantitative assessment of LV fibrosis for risk stratification of patients with coexisting AF and HF.

## Supplementary information

Supplementary Tables

## Data Availability

The datasets generated during and/or analysed during the current study are available from the corresponding author on reasonable request.
