# Supplementary material for: Prognostic Significance of Left Ventricular Fibrosis Assessed by T1 Mapping in Patients with Atrial Fibrillation and Heart Failure
Source: Sci Rep. 2019 Sep 16;9:13374. doi: 10.1038/s41598-019-49793-8 (PMC6746785; doi:10.1038/s41598-019-49793-8)
Supplement: Supplementary file 1 — Supplementary Tables [file 41598_2019_49793_MOESM1_ESM.pdf]

Prognostic Significance of Left Ventricular Fibrosis Assessed by T1 Mapping in Patients with Atrial Fibrillation and Heart Failure

Author list:

Lei Zhao, MD; Songnan Li, MD; Xiaohai Ma, MD; Rong Bai, MD; Nian Liu, MD; Ning Li, MD; Paul Schoenhagen, MD; Changsheng Ma, MD

Supplementary Table S1

Univariable and Multivariable Analysis of Variables Predicting Composite Adverse Outcome in Patients with Left Ventricular Interstitial Fibrosis

Variable	Univariate Analysis Unadjusted HR (95% CI)	p Value	Multivariable Analysis Adjusted HR (95% CI)	p Value
Age	1.023 (0.993-1.054)	0.139		
Female	1.849 (0.814-4.204)	0.142		
BMI	1.009 (0.893-1.139)	0.890		
Duration of AF	1.002 (0.997-1.008)	0.411		
AF patterns	1.832 (0.871-3.863)	0.110		
Hypertension	1.482 (0.600-3.660)	0.394		
Diabetes mellitus	4.032 (1.192-13.635)	0.025	1.133 (0.289-4.443)	0.858
CHA_2DS_2-VaSc score	1.215 (0.949-1.556)	0.122		
LA volume index	1.028 (1.013-1.044)	<0.001	1.015 (0.993-1.037)	0.192
SR_e	0.970 (0.948-0.992)	0.008	0.974 (0.921-1.031)	0.361
LV EF	0.955 (0.927-0.984)	0.002	1.046 (0.962-1.138)	0.289
LV $ECV_{average}$ (per 1% increase)	1.425 (1.246-1.630)	<0.001	1.399 (1.167-1.678)	<0.001

Supplementary Table S2

Univariable and Multivariable Analysis of Variables Predicting Heart Failure Readmission in Patients with Left Ventricular Interstitial Fibrosis

Variable	Univariate Analysis Unadjusted HR (95% CI)	p Value	Multivariable Analysis Adjusted HR (95% CI)	p Value
Age	1.012 (0.979-1.046)	0.488		
Female	2.129 (0.867-5.231)	0.099		
BMI	0.968 (0.846-1.107)	0.633		
Duration of AF	1.003 (0.997-1.009)	0.361		
AF patterns	1.775 (0.768-4.102)	0.179		
Hypertension	1.191 (0.402-3.525)	0.752		
Diabetes mellitus	4.759 (1.383-16.375)	0.013	1.242 (0.305-5.062)	0.762
LA volume index	1.037 (1.019-1.051)	<0.001	1.019 (0.993-1.045)	0.153
SR_e	0.958 (0.933-0.984)	0.002	0.970 (0.907-1.037)	0.369
LV EF	0.942 (0.912-0.973)	<0.001	1.041 (0.946-1.146)	0.411
LV $ECV_{average}$ (per 1% increase)	1.482 (1.273-1.726)	<0.001	1.384 (1.128-1.699)	0.002
